# Thyroid auto‐antibodies in newly diagnosed multiple sclerosis patients: A cross sectional study

**DOI:** 10.1002/hsr2.2247

**Published:** 2024-07-09

**Authors:** Maryam Poursadeghfard, Arashk Mallahzadeh, Ava Hamidi, Maryam Owjfard

**Affiliations:** ^1^ Clinical Neurology Research Center Shiraz University of Medical Sciences Shiraz Iran

**Keywords:** anti‐thyroglobulin antibody, anti‐thyroid peroxidase antibody, multiple sclerosis, thyroid stimulating hormone

## Abstract

**Introduction:**

Multiple sclerosis (MS) is a chronic inflammatory disease in which the immune system attacks the myelin sheath of the central nervous system (CNS). It has been proposed that autoimmune conditions may occur together and an individual's immune system may attack more than one system. Autoimmune thyroid disease is one of the most common comorbidities along with MS. Since thyroid hormones are crucial for normal brain function and remyelination, we aimed to determine the prevalence of thyroid dysfunction in a group of MS patients compared with healthy controls.

**Methods:**

This cross‐sectional study was conducted in medical clinics affiliated to Shiraz University of Medical Sciences, South of Iran. To prevent the effects of MS modifying drugs on thyroid function, we examined 73 newly diagnosed MS patients, which had not been treated yet, compared to 72 healthy individuals.

**Results:**

After measurement of the serum level of TSH, Anti TPO‐Ab, and Anti TG‐Ab, we found a significantly higher prevalence rate of abnormal TSH levels (high or low) in the MS group (*p* = 0.02). We also found a higher frequency of thyroid dysfunction in the female MS group (*p* = 0.01). However, there was no significant difference in the two other anti‐thyroid antibodies among the groups. Our results demonstrate a significant and positive linear relationship between age and TSH levels (*R* = 0.402; *p* < 0.001) and also age and Anti TPO‐Ab levels (*R* = 0.397; *p* < 0.001) among the MS population.

**Conclusion:**

We found a higher prevalence of TSH alteration among the MS population. Anti TPO‐Ab and Anti TG‐Ab levels did not differ among groups. These findings suggest that MS patients might be at an increased risk for thyroid dysfunction. However, further studies are required to determine the underlying cause. The linear relationship between age and TSH and Anti TPO‐Ab levels in MS patients suggest that there is an association between TSH dysfunction and age.

## 1 BACKGROUND

MS is an inflammatory disease of the CNS that damages both oligodendrocytes and axons leading to diverse symptoms.^1^ The exact cause of this disease is not yet determined, however numerous findings point to the roles of both genetic predisposition and environmental factors and the disease has been generally accepted as an autoimmune condition.^2^

Studies have shown that people with one autoimmune disease are susceptible to developing another one and that autoimmune diseases may occur together.^3^ About 25% of MS patients are diagnosed with at least one other autoimmune disorder. Hashimoto thyroiditis, psoriasis, irritable bowel disease, and rheumatoid arthritis are some of the comorbidities with MS.^4^, ^5^

A notable correlation exists between autoimmune thyroid diseases (ATD) and MS, affirming the heightened risk of thyroid disorders in individuals with MS.^6^ Thyroid‐mediated signaling is essential in the process of myelination and remyelination and thyroid hormone administration has been shown to improve myelin repair.^7^ Due to the important role of thyroid hormones in the normal process of myelin repair, many studies have investigated the prevalence of thyroid dysfunction in MS patients.

Other than that, fatigue, muscle weakness, and myalgia are common symptoms in both MS and hypothyroidism,^8^ therefore a co‐existing ATD, which is the most common cause of hypothyroidism could be missed. That being said, due to the common symptoms in both MS and ATD and the role of thyroid hormones in remyelination, it is crucial to evaluate thyroid function when MS patients present with such symptoms.

To better understand any correlation between MS and ATD we conducted this study in Shiraz, south of Iran, to compare the prevalence of autoimmune thyroid auto‐antibodies in MS patients with the control group. To prevent the effects of MS‐modifying drugs on thyroid function, we selected our patients among those who had not yet received disease‐modifying treatments for MS. We examined serum levels of Thyroid Stimulating Hormone (TSH), Anti Thyroid Peroxidase Antibody (Anti TPO‐Ab) and Anti‐Thyroglobulin Antibody (Anti TG‐Ab) in MS patients and the control group.

## 2 METHODS

### 2.1 Study design and patients

The present study is a cross‐sectional study that was conducted in a descriptive and analytical manner. All experiments were accepted by the Ethics Committee of Shiraz University of Medical Sciences (SUMS). In this study, we investigated the prevalence of autoimmune thyroid diseases in patients with MS. The research participants included all newly diagnosed MS patients based on McDonald's 2017 MS diagnosis criteria and who had not received any treatment for MS except methylprednisolone ^9^ and were referred to all medical centers affiliated to SUMS, Shiraz, Iran, within a period of 6 months. The control group was the patients who had referred to these centers due to headaches during this period and who had no history of any other disease. Written informed consent was obtained from all individuals enrolled in this study.

### 2.2 Data collection

Patient information was collected using a checklist. The checklist consists of two parts. The first part is related to demographic characteristics, including age, sex, marital status, etc. The second part is related to the patient's clinical and paraclinical information, including the serum level of Anti TPO‐Ab and Anti TG‐Ab and TSH.

### 2.3 Blood samples

We referred the MS patients and the control group to the laboratory. After an overnight fasting, venous blood samples were taken and centrifuged at 3,000 rpm for 10 min, and samples were stored at −80°C until analysis. The concentration of anti‐thyroid antibodies (Anti‐TPO or Anti‐TG) and TSH were measured with the immunochemiluminometric assay (ICMA). The reference ranges were 0.3–5.3 µIU/mL for TSH, 0–35 IU/mL for Anti‐TPO, and 0–115 IU/mL for Anti‐TG. One‐step Immunoradiometric assay (IRMA) was used for measuring TSH as recommended by manufacturers (Siemens).

### 2.4 Statistical analysis

Data were analysed using SPSS version 26.0 (IBM, New York, NY, USA). All results were presented as median and interquartile range (IQR) for quantitative variables and frequency (%) for qualitative variables. The Kolmogorov–Smirnov test was used to check for normality of distribution. The differences in categorial variables including gender, abnormal or normal levels of TSH, Anti‐TG, and Anti‐TPO were assessed using the two‐tailed Pearson's Chi‐square test. The two‐tailed Mann‐Whitney U test was used for age difference. Pearson's correlation coefficient was used to analyze the relationship between age and serum TSH/Anti TPO‐Ab/Anti TG‐Ab levels. *p* < 0.05 was used to indicate a significant difference. The results were interpreted and reported following the guidelines set forth by Assel et al.^10^

## 3 RESULTS

A total of 72 patients with MS and 73 healthy controls have been evaluated.

The median age of subjects was 30, IQR:25‐35. The median age of the MS patients was 30, IQR: 25–35, and the median age of the control group was 31, IQR: 27–35. We found no significant difference in age among the participants (*p* = 0.28).

Among the participants, 96 people (66.2%) were women and 49 people (33.8%) were men. The frequency of females in the MS patient group was 74% and, in the control group was 58.3%. The difference in gender between the control group and the patients was statistically significant (*p* = 0.04).

Among MS patients, 17 individuals (23.3%) displayed total abnormal TSH levels—either higher or lower than the normal range. In contrast, 7 individuals (9.7%) in the control group showed abnormal TSH levels. This difference was statistically significant (*p* = 0.02).

Although we found that a higher percentage of MS patients had abnormal Anti‐TG (8.2%) and abnormal Anti‐TPO (16.4%) compared to the control group, difference among groups did not meet standard statistical significance (see Table 1).

**Table 1:** Serum level of TSH, Anti TG‐Ab and Anti TPO‐Ab in the MS patients and control groups.

Variable		MS patients' group	Control group	*p*‐value
TSH	Abnormal (<0.3)	15 (20.5%)	2 (2.8%)	
	Abnormal (>5.3)	2 (2.7%)	5 (7.0%)	
	Total abnormal	17 (23.3%)	7 (9.8%)	0.02
	Normal	56 (76.7%)	64 (90.1%)	
Anti TG‐Ab	Normal	67 (91.8%)	67 (93.1%)	0.77
	Abnormal	6 (8.2%)	5 (6.9%)	
Anti TPO‐Ab	Normal	61 (83.6%)	64 (88.9%)	0.35
	Abnormal	12 (16.4%)	8 (11.1%)	

Table 2 shows subgroup analysis of TSH, Anti TG‐Ab, and Anti TPO‐Ab in female patients with MS compared with the female control group. According to the results, the frequency of abnormal TSH levels in female patients with MS was more than that of females in the control group, and this observed difference was statistically significant (*p* = 0.01). The proportion of abnormal Anti TG‐Ab in females from the control group exceeded that of female patients with MS. Conversely, the percentage of abnormal Anti TPO‐Ab in female MS patients surpassed that of females in the control group, although no significant differences were observed (*p* = 0.96, *p* = 0.51 respectively).

**Table 2:** Serum level of TSH, Anti TG‐Ab and Anti TPO‐Ab in the female MS patients and control groups.

Variable		MS patients' group	Control group	*p*‐value
TSH	Abnormal (<0.3)	10 (18.5%)	1 (2.4%)	
	Abnormal (>5.3)	2 (3.7%)	1 (2.4%)	
	Total abnormal	12 (22.2%)	2 (4.8%)	0.01
	Normal	42 (77.8%)	40 (95.2%)	
Anti TG‐Ab	Normal	49 (90.7%)	38 (90.5%)	0.96
	Abnormal	5 (9.3%)	4 (9.5%)	
Anti TPO‐Ab	Normal	45 (83.3%)	37 (88.1%)	0.51
	Abnormal	9 (16.7%)	5 (11.9%)	

Table 3 demonstrates comparison of TSH, Anti TG‐Ab, and Anti TPO‐Ab in the male MS patients group compared to the male control group. According to the results, the percentage of abnormal TSH in male patients with MS was more than that of men in the control group (26.3% vs 16.6%), but the differences were not statistically significant (*p* = 0.41). The percentage of both abnormal Anti TG‐Ab, and Anti TPO‐Ab of males with MS was higher than the control group but the differences observed were not statistically significant (*p* = 0.73, *p* = 0.54 respectively).

**Table 3:** Serum level of TSH, Anti TG‐Ab, and Anti TPO‐Ab in the male MS patients and control groups.

Variable		MS patients' group	Control group	*p*‐value
TSH	Abnormal (<0.3)	5 (26.3%)	1 (3.3%)	
	Abnormal: (>5.3)	0 (0.0%)	4 (13.3%)	
	Total abnormal	5 (26.3)	5 (16.6)	0.41
	Normal	14 (73.7%)	25 (83.3%)	
Anti TG‐Ab	Normal	18 (94.7%)	29 (96.7%)	0.73
	Abnormal	1 (5.3%)	1 (3.3%)	
Anti TPO‐Ab	Normal	16 (84.2%)	27 (90.0%)	0.54
	Abnormal	3 (15.8%)	3 (10.0%)	

**Figure 1:**
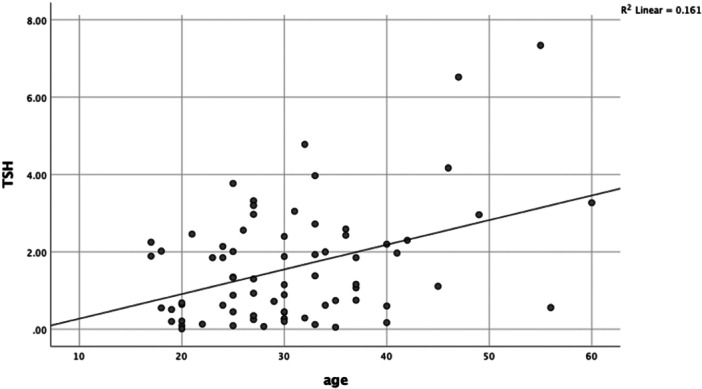
Correlation between age and TSH concentration in the MS group. There is a linear and positive correlation between the age of the patients and their TSH concentration (*R* = 0.402, *p* < 0.001).

**Figure 2:**
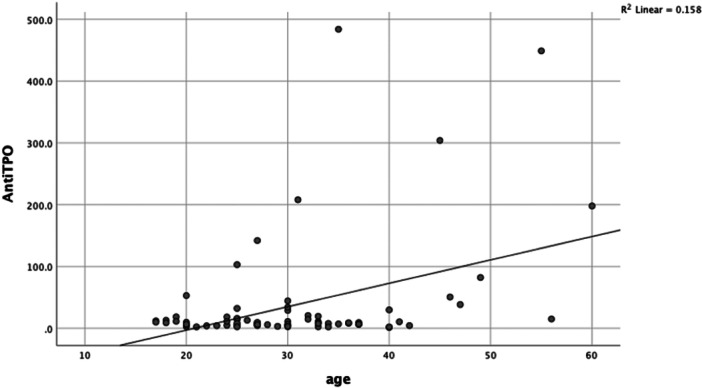
Correlation between age and Anti TPO‐Ab concentration in the MS group. There is a linear and positive correlation between age and serum Anti TPO‐Ab in the MS group (*R* = 0.397, *p* < 0.001).

Pearson's correlation analysis between age and TSH levels/Anti TPO‐Ab in the MS patients and control groups showed that there were significant linear and positive relationship between the age of the patients and their TSH (*R* = 0.402; *p* < 0.001) and Anti TPO‐Ab (*R* = 0.397; *p* < 0.001) levels (Figures 1 and 2). However, this relationship was weak and not significant in the control groups (*R* = 0.091; *p* = 0.44), (*R* = 0.095; *p* = 0.42) respectively.

The correlation analysis between age and Anti TG‐Ab levels in the MS patients showed a nonsignificant, weak linear and inverse correlation (*R* = ‒0.004; *p* = 0.48). Moreover, no significant correlation was found for the control group (*R* = 0.037; *p* = 0.37).

## 4 DISCUSSION

Among the many comorbidities that may be observed with MS, ATD is one of the most common autoimmune conditions reported in MS patients.^11^ However, in some instance, thyroid dysfunction seems to be a result of disease modifying therapies (DMT) such as alemtuzumab and interferon‐beta.^12^, ^13^ It is important to evaluate whether thyroid dysfunction in MS patients is independent from DMT and to establish its true cause among these individuals.^14^ The most common finding in MS patients is a positive Anti TPO‐Ab with a normal functioning thyroid.^15^ Previous studies have examined thyroid function among the MS population. Results of these studies vary, some studies found no thyroid dysfunction among MS and control groups while some studies demonstrated an increased rate of thyroid dysfunction in MS patients which will be discussed further.^6^ In the present study, we measured TSH levels, along with Anti‐TG Ab and Anti‐TPO Ab. According to the findings of previous studies, the prevalence of thyroid dysfunction in MS patients ranges between 6.44% and 11%.^11^, ^16^ In our study, we found that 23.3% of MS patients had abnormal TSH levels which was significantly higher than the control group (9.8%). Our results demonstrated that among genders, TSH abnormality is higher in both male and female MS group compared to the same gender of the control group. Although this difference in men was not statistically significant. Autoimmunity is considered the most frequent etiology of thyroid dysfunction. A very important part of the genetic contribution to autoimmunity is gender.^17^ L Durelli et al. demonstrated that high or low TSH levels did not differ between MS and the control group and MS patients are not at an increased risk for abnormal TSH levels even if antithyroid microsomal antibodies were positive which differs from the results of our study.^18^

A study conducted by Annunziata et al. evaluated the difference of anti‐thyroid antibodies (Anti TPO‐Ab and Anti TG‐Ab) among MS, normal healthy (NH), and other neurological disease (OND) subjects.^19^ Anti‐TPO was significantly higher in MS patients (21.7%) compared to NH (5.3%) and OND subjects (9.2%). TG is a large glycoprotein and consists of an average of 2–3 molecules of T4 and 0.3 molecules of T3.^20^ Autoantibodies of TPO and TG are associated with ATD.^21^, ^22^ We found no significantly different rates of abnormal Anti‐TPO in MS patients (Table 1) and males and females did not differ in any of the anti‐thyroid antibodies in our study (Tables 2, 3) which differs from the findings of Annunziata et al. These diverse results could be affected by several factors. Some studies lack a control group and since iodine deficiency is associated with lower rates of anti‐thyroid antibodies, it is necessary to consider iodine sufficiency when conducting these studies.^15^

### 4.1 limitations and strengths

our study has some limitations. The number of included participants is quite low. Due to financial issues, T3 and T4 hormone levels could not be measured among participants and we had to rely on TSH levels and were unable to exactly categorize patients as hypo or hyperthyroidism. Furthermore, socioeconomic differences can impact the study results since socioeconomic factors greatly influence both thyroid disorders and multiple sclerosis.^23^, ^24^ Lower socioeconomic individuals face barriers in accessing healthcare services which could mean that individuals with lower socioeconomic status are less likely to receive timely diagnoses of MS or thyroid dysfunction. Moreover, although it hasn't been fully cleared, some studies suggest that the incidence of MS is higher among high socioeconomic individuals.^23^ Future studies should consider the role of socioeconomic factors since it may affect the generalizability of findings across different socioeconomic groups.

Difference in technological infrastructure should be considered since it could misguide researchers when comparing results of thyroid function with other studies.

Strengths of this study include that this is a case‐control study conducted in an iodine‐efficient area. Most studies that evaluated thyroid function in MS individuals have not considered the role of DMT on thyroid function. In the present study, we included newly diagnosed MS patients who haven't received DMT yet, and their thyroid function tests have not been affected by other drugs.

## 5 CONCLUSION

In summary, our findings show a higher prevalence of abnormal TSH (high or low) in MS patients. Female MS patients were at increased risk of this abnormality. Anti TPO‐Ab and Anti TG‐Ab did not differ among groups. Our study was based on MS patients who haven't received disease modifying treatment and conducted on a noniodine deficient population which can affect thyroid function tests and anti‐thyroid antibody levels. Further studies are needed to provide information about the co‐occurrence of auto immune thyroid disease with MS.

## AUTHOR CONTRIBUTIONS

**Maryam Poursadeghfard**: Conceptualization; Investigation; Methodology; Data curation; Funding acquisition. **Arashk Mallahzadeh**: Investigation; Writing—original draft; Writing—review & editing; Data curation; Formal analysis. **Ava Hamidi**: Writing—review & editing; Project administration; Data curation; Investigation. **Maryam Owjfard**: Investigation; Writing—review & editing; Supervision; Data curation.

## TRANSPARENCY STATEMENT

The lead author Maryam Owjfard affirms that this manuscript is an honest, accurate, and transparent account of the study being reported; that no important aspects of the study have been omitted; and that any discrepancies from the study as planned (and, if relevant, registered) have been explained.

## Data Availability

The data that support the findings of this study are available from the corresponding author upon reasonable request.
